# Biomarkers in External Apical Root Resorption: An Evidence-based Scoping Review in Biofluids

**DOI:** 10.5041/RMMJ.10482

**Published:** 2022-10-27

**Authors:** Priyanka Kapoor, Aman Chowdhry, Dinesh Kumar Bagga, Deepak Bhargava

**Affiliations:** 1 School of Dental Sciences, Sharda University, Greater Noida, Uttar Pradesh, India; 2 Department of Orthodontics, Faculty of Dentistry, Jamia Millia Islamia, New Delhi, India; 3 Department of Oral Pathology and Microbiology, Faculty of Dentistry, Jamia Millia Islamia, New Delhi, India; 4 Department of Orthodontics and Dentofacial Orthopaedics, School of Dental Sciences, Sharda University, Greater Noida, Uttar Pradesh, India; 5 Department of Oral Pathology and Microbiology, School of Dental Sciences, Sharda University, Greater Noida, Uttar Pradesh, India

**Keywords:** Biomarkers, gingival crevicular fluid, interleukin, orthodontics, root resorption

## Abstract

**Background:**

External apical root resorption (EARR), an unwanted sequela of orthodontic treatment, is difficult to diagnose radiographically. Hence, the current scoping review was planned to generate critical evidence related to biomarkers in oral fluids, i.e. gingival crevicular fluid (GCF), saliva, and blood, of patients showing root resorption, compared to no-resorption or physiologic resorption.

**Methods:**

A literature search was conducted in major databases along with a manual search of relevant articles in the library, and further search from references of the related articles in March 2021. The initial search was subjected to strict inclusion and exclusion criteria according to the Preferred Reporting Items for Systematic Reviews and Meta-Analysis (PRISMA) guidelines.

**Results:**

Following PRISMA guidelines, 20 studies were included in the final review. The studies included human clinical trials and cross-sectional and prospective studies with/without control groups with no date/language restriction. Various biomarkers identified in EARR included dentinal proteins, enzymes, cytokines, and salivary proteins. Severe resorption had higher dentin sialoprotein (DSP) and resorption protein concentrations as well as lower granulocyte-macrophage colony-stimulating factor (GM-CSF) as compared with mild resorption. Increased DSP and dentin phosphophoryn (DPP) expression was found in physiologic resorption. Compared to controls, resorbed teeth showed a higher receptor activator of nuclear factor kappa B ligand/osteoprotegerin (RANKL/OPG) ratio. In contrast, levels of anti-resorptive mediators (IL-1RA, IL-4) was significantly decreased. Differences in force levels (150 g and 100 g) showed no difference in resorption, but a significant rise in biomarkers (aspartate transaminase [AST] and alkaline phosphatase [ALP]) for 150 g force. Moderate to severe resorption in young patients showed a rise in specific salivary proteins, requiring further validation. Limitations of the studies were heterogeneity in study design, biomarker collection, sample selection, and confounding inflammatory conditions.

**Conclusions:**

Various biomarkers in biofluids indicate active resorption, while resorption severity was associated with DSP and GM-CSF in GCF, and a few salivary proteins. However, a robust study design in the future is mandated.

## INTRODUCTION

External apical root resorption (EARR) may occur in a variety of conditions like bacterial invasions, trauma, neoplasms, and systemic or pressure conditions produced by the application of orthodontic forces.^1^ External apical root resorption is also known as orthodontically induced root resorption (OIRR), which is an undesirable but common sequela of orthodontic tooth movement.^2^,^3^

External apical root resorption has a multifactorial etiology and is associated with several risk factors predisposing patients to various degrees of root resorption.^2^,^3^ The reported EARR incidence is variable: 90% in histological studies, 73% in radiological studies after tooth movement, 6%–13% depending on the type of teeth, and 1%–5% or 1%–2% depending on resorption severity.^3^,^4^ Nevertheless, any grade of EARR severity is known to limit the outcome of successful orthodontic treatment and also cause oral dysfunction on progression.^5^

The deleterious effects of EARR on tooth movement mandate early detection of resorption. However, early detection is not possible with the currently available diagnostic modalities that rely on two- or three-dimensional radiographs.^3^,^6^ Radiographs are associated with limitations such as radiation exposure, inability to outline the active resorption process, and limited view and standardization of the resorption process.^3^,^5^,^7^ Hence, there is a great need for non-invasive techniques or determination of biomarkers to detect root resorption early in susceptible patients.^5^

**Figure 1:**
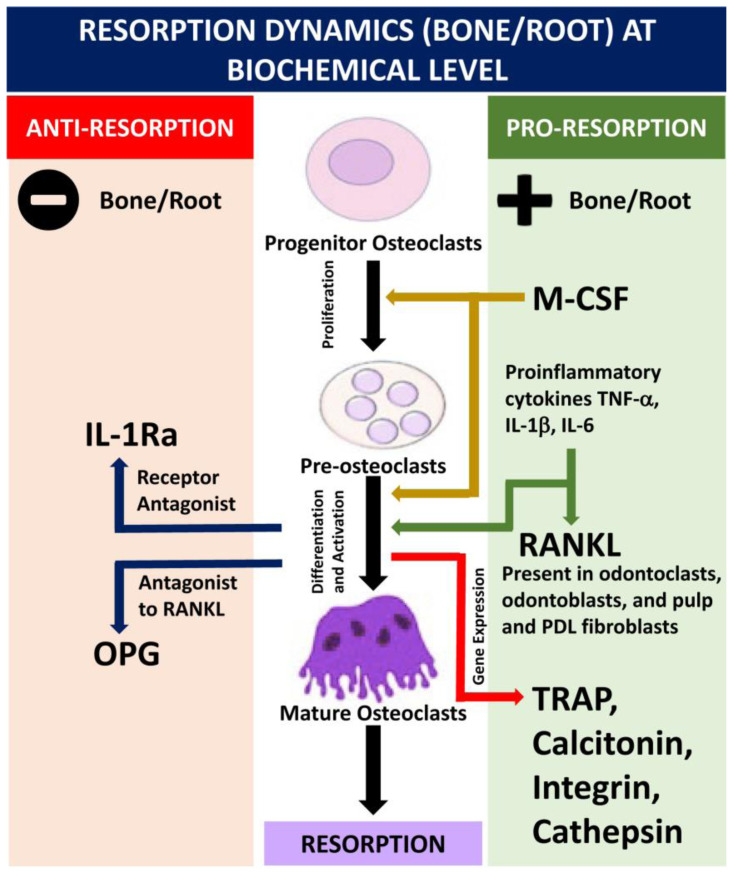
Diagrammatic Representation of Resorption Dynamics at the Biochemical Level. The bone/root resorption process is similar at cellular levels for both osteoclasts and odontoclasts.^8^ Cellular differentiation from mononucleated (progenitor osteoclasts) to multinucleated (mature osteoclasts) involves pro-resorption and anti-resorption. The M-CSF is responsible for proliferation and differentiation of progenitor cells; proinflammatory cytokines are directly responsible for differentiation and activation of pre-osteoclasts, or activation of RANKL, which in turn leads to pro-resorptive gene expression (TRAP, etc.). Anti-resorptive OPG (decoy receptor for RANKL) and IL-1RA (receptor antagonist to IL-1β) govern the resorptive activity, with the RANKL/OPG ratio being the primary governing factor. IL-1β, interleukin-1β; IL-1RA, interleukin-1 receptor antagonist; IL-6, interleukin 6; M-CSF, macrophage colony-stimulating factor; OPG, osteoprotegerin; PDL, periodontal ligament; RANKL, receptor activator of nuclear factor kappa-B ligand; TNF-α, tumor necrosis factor-α; TRAP, tartrate-resistant acid phosphatase.

To define the biomarkers in root resorption, a thorough understanding is needed of its pathophysiology in relation to the surrounding bone and the periodontal ligament housing different types of cells, matrix, and biological messengers,^8^,^9^ as explained in Figure 1. Although the biomarkers released in the paracrine environment in the gingival crevicular fluid (GCF) have been extensively studied in bone resorption during orthodontic tooth movement,^10^,^11^ a comprehensive study of all body fluid biomarkers (GCF, saliva, and blood) around teeth undergoing resorption is lacking. Various mediums have been evaluated for biomarker collection, of which GCF has the advantages of ease of repeatability, collection, and detection of early resorption.^5^ Also, saliva has greater accessibility and ease, but is comparatively less specific to the underlying periodontal condition.^12^

Various biomarkers are indicative of active resorption, with evidence supporting the presence of dentinal proteins including dentin sialophosphoprotein (DSPP), dentin sialoprotein (DSP), and dentin phosphophoryn (DPP) in GCF and saliva.^5^,^7^,^13^ Of these, DSPP shows a continuous expression in amelogenesis and dentinogenesis^14^ and is considered a potent resorption marker. Other markers responsible for osteoclastogenesis or extracellular matrix degradation, including pro-inflammatory cytokines (interleukins [IL], tumor necrosis factor, etc.) or matrix metalloproteinases (MMPs), have also been associated with the degree of resorption.^9^,^15^,^16^ Alkaline phosphatase (ALP), an enzyme associated with early deposition of minerals and tissue calcification, may contribute toward pulpal repair and healing after traumatic insults or injury and shows variable expression in root resorption.^15^

Hence, there are multiple mediators having distinct associations in resorption, some in tissue destruction and others in tissue repair, which show variable expression at different stages of resorption. The success of clinical orthodontic treatment in turn is dependent on early detection of EARR and on preventing and limiting the extent of this unwanted condition. Tarallo et al. have provided some evidence related to the role of GCF biomarkers in root resorption, but failed to establish an all-inclusive understanding of the dynamics of root resorption markers to identify the most potent biomarker that might show significant association in multiple oral biofluids.^17^ Another study by Allen et al. examined salivary protein in orthodontic tooth movement, but it did not specifically target root resorption.^18^

Hence, this scoping review addresses the gap in the literature to generate critical evidence related to biomarkers in all oral fluids (gingival crevicular fluid [GCF], saliva, blood) of patients showing root resorption, compared to no resorption or physiologic resorption.

## MATERIAL AND METHODS

### Protocol

A scoping review was designed according to the Preferred Reporting Items for Systematic Reviews and Meta-analysis (PRISMA) guidelines specific to scoping reviews (PRISMA-ScR). The inclusion and exclusion criteria were defined (Table 1), and the study was registered in the Open Science Framework (https://osf.io/nep9z/). No funding was received for the study.

**Table 1:** Inclusion and Exclusion Criteria for Study Selection.

Inclusion Criteria	Exclusion Criteria
All original studies on humans including clinical trials Prospective or retrospective cohort studies Studies mentioning both biomarkers and root resorption Studies in orthodontics and physiological root resorption	*In vitro* studies Animal studies Studies on biomarkers but not on resorption Studies on resorption but not on biomarkers Case reports and reviews/opinions

### Eligibility

The research topic for determining literature eligibility was developed based on the PICOS model, as follows: Population, patients showing EARR on radiographs; Intervention, orthodontic forces; Comparison, no resorption or physiological resorption; Outcomes, change in biomarkers in biofluids. There was no limitation of date or language placed on the literature search.

Based on the above, the research question asked: was the variation in levels of biomarkers in the oral fluids associated with root resorption in patients undergoing orthodontic treatment in comparison to no resorption or physiologic resorption?

### Information Sources and Search

In March 2021, a thorough literature search was conducted in the major databases: PubMed, Web of Science, J-Gate, Directory of Open Access Journals, Scopus, and Embase, along with related searches, manual searches, and tracking of references from the manual searches. Both MeSH and free-text terms were used to search most of the databases: “biomarkers,” “root resorption,” and “orthodontics” with the BOOLEAN terminology “AND.” Duplicate results were removed.

### Study Selection

**Figure 2:**
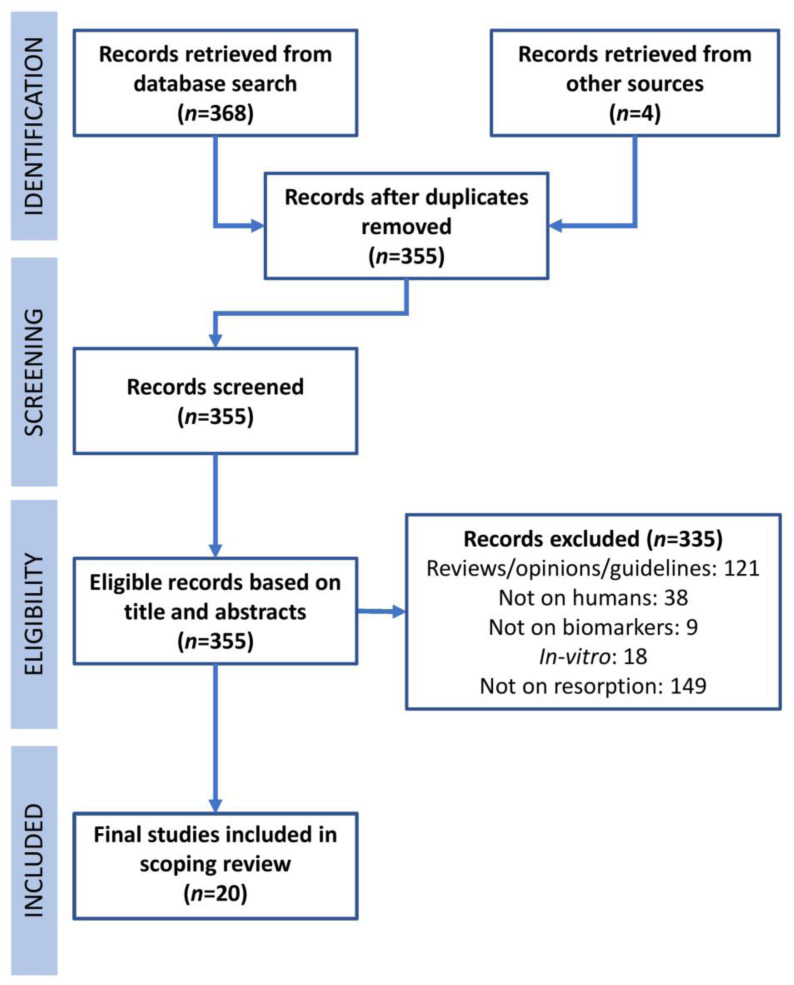
PRISMA (Preferred Reporting Items for Systematic Reviews and Meta-Analysis) of Evidence Related to Biomarkers in EARR Based on Predetermined Search Strategy.

The identification, screening, eligibility, and inclusion of studies were performed as detailed in the PRISMA flowchart shown in Figure 2. The search strategy was applied independently by two reviewers (PK and AC) strictly based on the inclusion and exclusion criteria (Table 1).

Any discordance was addressed by two reviewers (DKB and DB) for a final consensus. Duplicates were removed, and articles were screened based on their titles and abstracts. Full texts were then retrieved, and an in-depth review was performed to identify the final studies selected for this review. No quality assessment was done as it is not mandatory for scoping reviews, and the aim of the current scoping review was to present a broad scope of biomarkers identified to date in EARR. Studies related to root resorption by other causes, including traumatic forces or endodontic resorption, were excluded from the final selection. The primary outcome included the variation in expression of different biomarkers in root resorption, which was further correlated with their mechanism in the cellular remodeling process.

### Data Charting

The data charting of these articles was performed by two investigators (PK and AC) independently, and any discordance was addressed by a third researcher (DKB). The criteria for data charting were according to JBI (Joanna Brigg’s Institute) based on author, reference, and primary outcomes or results relevant to the broad research question.^17^

## RESULTS

### Study Selection

A total of 372 articles were initially identified, duplicate publications were removed, and the inclusion and exclusion criteria were applied, resulting in 20 articles found being included in the final review (Figure 2).^2^,^5^,^7^,^9^,^12^,^13^,^15^,^16^,^19^–^30^

### Data Extraction

The data extraction from each study related to participant and study characteristics, the biomarker(s) studied, the medium and technique of biomarker(s) study, and the outcomes related to biomarker(s) expression. Full details are given in Table 2.

**Table 2:** Evidence Related to Study of Various Biomarkers and Proteins in Various Biofluids Associated to EARR.

Authors	Experimental Subjects/Teeth (No./Age/Sex)	Biomarkers Studied	Condition Analyzed	Detection of Root Resorption	Controls/Teeth (No./Age/Sex)	Medium Studied	Technique	Outcomes	Conclusions
2004 Mah et al.^13^	Grp 1: Mx central incisor (*n*=20) with 1–3 mm RR; 13F, 7M; 12–16 y Grp 2: 10 second molars (*n*=20); 15F, 5M; 9–12 y	DPP	Orthodontic Tx (not specified)	Radiographs (not specified)	Mx central incisors (*n*=20) of untreated pts, 12F, 8M, 12–16 y	GCF	Periopaper, ELISA	Levels of DPP: greatest in resorbing 1^0^ molar (11.7±4.1 μg/mg) followed by orthodontically treated tooth (9.3±4.7 μg/mg) and least in controls (5.4±4.1 μg/mg); NS between resorption Grps	DPP can be detected in exfoliating primary teeth and orthodontic root resorption
2007 Balducci et al.^7^	20 pts with mild RR (≤2 mm) (11F, 9M, 14–40 y), 20 pts with severe RR (>2 mm) (15F, 5M, 15–44 y)	DMP1, PP, DSP	RR in orthodontic pts	IOPA	20 pts (13F, 7M, 12–34 y); no RR/orthodontic Tx	GCF	Periopaper (mesial and distal of Mx central and lateral incisors), SDS–PAGE, stained western blot, ELISA	Molecular weight 77, 66, 55, 50, and 26 kDa proteins identified, NS between control and study Grps in immunoblot; ELISA showed Sig. ↑ of DMP1, PP, DSP in RR vs control Grps and of PP and DSP in severe RR vs mild RR Grps	DMP1, DSP, and PP in GCF proved a biomarker for RR in orthodontic Tx
2008 Kereshanan et al.^5^	Grp 1: 50 second 1^0^ molars (9–14 y) (advanced coronal RR [*n*=33] and apical minimal RR Grp [*n*=17]) Grp 2: 20 pts (11–15 y), T0=pre-fixed Tx, T1=12 mo post start of Tx	DSP	Physiological RR and OTM	Orthopantomogram	Control: 20 pts (10–15 y) erupted second premolars with no RR	GCF	Micropipettes, slot blot immunoassay, DSP in dentin of 1^0^ molars by western blot	DSP levels: greater in physiological RR than non-resorbing teeth, DSP levels NS between coronal RR and apical RR; DSP levels Sig. higher in T1 compared to T0	DSP in GCF proved a biomarker of root resorption
2009 George et al.^19^	Grp 1: mild RR of 2 mm (20 pts, Tx 1 y) Grp 2: severe RR >2 mm (20 pts)	OPN, OPG, RANKL	Orthodontic Tx (not specified)	Radiographs (not specified)	20 pts: no Tx, no RR	GCF	Periopaper (mesial/distal of Mx central and lateral incisors), SDS-PAGE, western blot	Proteins conc greater in severe RR (0.89 μg/μL ±0.32 μg) than mild RR (0.77 μg/μL ±0.21 μg) and least in controls (0.22 μg/μL ±0.05 μg); ELISA showed Sig. higher RANKL antibodies in RR Grps than control Grp; RANKL/OPG ratio in severe RR Sig. greater than in control Grp	Presence of OPN, OPG, and RANKL in root resorption
2013 Kunii et al.^9^	5 pts with severe RR (5F, mean age 28.9±6.1 y; mean orthodontic Tx duration of 27.8±3.3 mo)	IL-6	All 4 extr orthodontic Tx	Radiographs (not specified)	15 pts without RR (13F, 2M, mean age, 28.0±5.3 y; mean orthodontic Tx duration of 26.4±3.1 mo)	GCF	Periopaper (mesial/distal of Mx central and lateral incisors), ELISA	IL-6 protein levels Sig. ↑ in RR than non-RR Grp	IL-6 in GCF proved a root resorption biomarker in orthodontic Tx
2013 Wahab et al.^15^	12F (Mx canines as test teeth), 100 g/150 g force to either side, split mouth design	ALP	Class II div 1 malocc; upper 4/4 extr with retraction by NiTi coil spring	IOPA	Mand canine as control	GCF, collection weekly for 6 wk	Periopaper (mesial/distal of Mx canine, Mand canine), spectrophotometry at 405 nm	ALP at mesial sites peak at wk 1 showing Sig. diff with 100 g force; no RR for test/control teeth in 150/100 g force	Canine movement greater with 150 g than 100 g force and higher ALP at mesial sites with no RR
2014 Sha et al.^2^	20 pts (12F, 8M, 13–24 y), 8–12 mo of orthodontic Tx	DSPP	Orthodontic Tx (not specified)	Radiographs (not specified)	Same pts for both methods (ELISA with spectrophotometry and electrochemical detection)	GCF	Filter paper strip (mesial/distal sites of left and right Mx central incisors), ELISA	DSPP detection with spectrophotometric ELISA 10 times greater than with electrochemical detection. DSPP conc range NS between methods	DSPP can be sensitively and accurately detected in root resorption
2014 Vieira^20^	Total 60 pts (38F, 22M, 15–30 y with orthodontic Tx of 6 mo); Grp 2: 30 pts, mild to moderate RR	Proteins	Orthodontic Tx (not specified)	IOPA	Grp 1: 30 pts, no RR	GCF	Sterile absorbent paper cones, 2-DE gels, SDS-PAGE with isoelectric focusing	Greatest sharpness to detect protein bands with Milli-Q ultrapure ice-cold water, without GCF protein extraction	Protein extraction protocols tested for accuracy
2014 Rody et al.^21^	11 pts (7F, 4M, 10–11 y) second 1^0^ molars with RR in one quadrant; split mouth design	Proteins	No orthodontic Tx	Radiograph (not specified)	11 pts (7F, 4M, 10–11 y), permanent 1st molar on contralateral side with no RR	GCF	Periopaper (lingual side of 1^0^ and permanent molars), LC-MS, nano-flow LC system coupled to triple TOF 5600 MS	Total 37 RR proteins upregulated and 59 RR proteins downregulated	RR proteins upregulation and downregulation identified in RR
2015 Wahab et al.^22^	19 (13F, 6M), split mouth design, either 100 g or 150 g force	ALP, TRAP, AST	All 4 extr and retr	IOPA	Internal control (baseline)	GCF, Baseline (0 wk), 1–5 wk	Periopaper (mesial/distal of Mx right and left canine), spectrophotometry	100 g Grp: TRAP Sig. ↑ from baseline to 3–5 wk and slight rise of ALP, AST from baseline; 150 g Grp: ALP, TRAP activities ↑ slightly from baseline, AST Sig. ↑ in 5 wk	150 g force and 100 g force show NS difference in AST, ALP, or TRAP levels
2016 Lombardo et al.^23^	6 pts (5F, 1M), average age 14 y, 12 wks orthodontic Tx	DSP	Orthodontic treatment with Damon appliances	Radiographs (not specified)	Same pts for both methods (conventional ELISA vs DSP antibody-coated magnetic micro-beads prior to ELISA)	GCF	Mesial and Ds sites of Mx central and lateral incisors, sterile paper strips	Sig. diff between standard ELISA and micro-beads for DSP evaluation in early RR evaluation; results of micro-bead approach are more uniform and highly sensitive	Modified micro-bead approach is more reliable for early detection of RR for DSP evaluation
2016 Rody et al.^16^	11 pts (7F, 4M, 10–11 y), second 1^0^ molars with RR in one quadrant	IL-1β, IL-1RA, MMP-8, DSP, RANKL, OPG	No orthodontic Tx	Radiograph (not specified)	Permanent 1st molar on contralateral side with no RR	GCF	Lingual side of 1^0^ and permanent molars, Periopaper, immunoassay	NS in IL-1β, OPG, or MMP-9 between exp and control Grp; RANKL data unreliable; IL-1RA Sig. downregulated in RR	IL-1RA down-regulation in GCF from 1^0^ molars with root resorption
2017 Yashin et al.^12^	9 pts (mean age 23±2.9 y), moderate to severe RR	Cytokine profile in saliva	Finished orthodontic Tx within 2 y	Orthopantomograms	Pts with no RR	Blood and saliva	10 mL unstimulated saliva collected by expectoration, ELISA	Saliva: moderate to severe RR show Sig. ↑ in IL-7, IL-10, IL-12p70, and IFN-γ, Sig. ↓ in IL-4; blood: control group has higher osteocalcin and P1NP than RR	Saliva can be used for cytokine assessment in root resorption
2017 Kaczor-Urbanowicz et al.^24^	48 pts with RR (31F, 17M) Grp 1: moderate to severe RR young pts (11F, 6M); Grp 2: moderate to severe RR adult pts (7F, 4M); Grp 3: mild RR young pts (7F, 4M); Grp 4: mild RR adult pts (6F, 3M)	Proteins	Not specified	IOPA (Mx central and lateral incisors) at T0 (before bonding), T9 (9 mo after bonding)	24 pts without RR (13F, 11M) Grp 5: control young pts (7F, 6M); Grp 6: control adult pts (6F, 5M)	Saliva	Unstimulated whole saliva, 2D gel electrophoresis, quantitative mass spectrometry, western blot	772 proteins identified by qMS, 244 highly increased expression profile, Sig. ↑ in moderate to severe young RR Grp compared to controls and 58 proteins in the adult Grp	Salivary proteins associated with root resorption identified
2017 Thalanany et al.^25^	20 pts, 13–22 y; exp Grp: 10 pts undergoing orthodontic Tx	DSPP	Simultaneous intrusion and retr arch	Radiograph (not specified)	Control Grp: no orthodontic Tx	GCF, T0= before intrusion, T1=2 mo after intrusion	Mx right and left central and lateral incisors; microcapillary tubes, ELISA	Sig. ↑ in DSPP at T1 compared to T0	DSPP may be marker for root resorption
2017 Ahuja et al.^26^	8 (2F, 6M, age range 13.9–22.9 y) Split mouth design: test vs control sides	IL-1β, 2, 4, 5, 6, 7, 8, 10, 12, 13, INF-γ, TNF-α, GM-CSF	225 g buccal tipping force for 28 d on test side	Micro-CT	Contralateral teeth (control side)	GCF, Time points: 0 h (prior to force), 3 h, 1 d, 3 d, 7 d, 28 d	Periopaper, multiplex bead immunoassay	IL-1β: Sig. ↑ peak at days 1 and 7 but NS between test and control side; IL-4: ↑, peak days 1–3; IFN-γ: peak at 72 h; TNF-α: ↑ at 3 h, 28 d; IL-7 peak at 28 d; GM-CSF: immediate ↓, ↑ at 7 d, peak at 28 d; Comparison between low and high RR: GM-CSF show Sig. ↑ in low RR; Micro-CT: mesial, distal surface, and middle 3^rd^ showed sig. ↑ RR on test side teeth	Pro-resorptive cytokines (IL-7, TNF-α) ↑ in high orthodontic forces, anti- resorptive cytokines (GM-CSF) ↓ initially
2018 Zhou et al.^27^	8F pts with RR, mean age 22.25 y, Tx duration 22.37 mo	Metabolites	Both extr and non-extr Tx	Orthopantomograms	11F controls, mean age 24.27 y, Tx duration 21 mo	Saliva	Unstimulated saliva collected from occlusal space of right Mand molars without chewing for ~3 min	187 metabolites identified, including butyrate, propane-1,2-diol, α-linolenic acid (Ala), α-glucose, urea, fumarate, formate, guanosine, and purine	Difference in metabolites in saliva of RR pts can be detected by 1 HNMR-based metabolomics method
2020 Mohd Nasri et al.^28^	10 pts	Protein abundance	Mx and Mand fixed appliances	IOPA at T0 and T6 of Mx central incisors	None	GCF at T0 (pre-Tx), T1 (1 mo), T3 (3 mo), T6 (6 mo)	Mesial and Ds of Mx central incisors Periopaper, liquid chromatography-tandem mass spectrometry	Increased protein abundance of S100A9, immunoglobulin J chain; heat shock protein 1A, immunoglobulin heavy variable 4–34 and vitronectin at T1; protein abundance ↑ of thymidine phosphorylase at T3	Early RR protein markers identified
2021 Mandour et al.^29^	74 pts (3 Grps: 2 Tx, 1 control) Grp 1: orthodontic pts (1–3 mm RR); Grp 2: pediatric pts (lower second 1^0^ molars, physiologic RR)	IL-1RA, DSPP	Not specified	Radiograph (not specified)	Grp 3: control (no RR, no orthodontic Tx)	GCF	Endodontic paper points, ELISA	IL-1RA levels in controls greater than orthodontic pts, and least in pediatric Grp; DSPP levels in pediatric group higher than in orthodontic pts, and least in controls; IL-1RA cut-off for OIRR (≤432.6 pg/mL) and DSPP (≥7.33 pg/mL); DSPP reliability (100%) vs IL-1RA (80%)	IL-1RA, DSPP biomarkers for OIRR
2020 Zain et al.^30^	7 orthodontic pts, 2 samples taken at 3 and 6 mo into orthodontic Tx and 3 samples at 12 mo of orthodontic Tx	DSPP	Fixed orthodontic Tx	Not specified	3 non-orthodontic control samples	GCF	Mx central incisors, Periopaper, absorption spectroscopy	Control sample showed lower peak in absorption spectrum than exp sample (3, 6, 12 mo); spectrum proportional to Tx duration, 0.91 accuracy	Higher absorption spectrum of DSPP indicates higher resorption

↑, greater/increase; ↓, lower/decrease; 1 HNMR, hydrogen-1 nuclear magnetic resonance; 10, primary; ALP, alkaline phosphatase; AST, aspartate aminotransferase; conc, concentration; CT, computed tomography; d, day(s); diff, difference; DMP1, dentin matrix protein 1; DPP, dentin phosphophoryn; Ds, distal; DSP, dentin sialoprotein; DSPP, dentin sialophosphoprotein; EARR, external apical root resorption; ELISA, enzyme-linked Immunosorbent assay; exp, experimental; extr, extractions; F, female(s); GCF, gingival crevicular fluid; GM-CSF, granulocyte-macrophage colony-stimulating factor; Grp, group(s); h, hour(s); IFN-γ, interferon gamma; IL, interleukin; IL-RA, interleukin-1 receptor antagonist; IOPA, intraoral periapical radiograph; LC, liquid chromatography; LC-MS, liquid chromatography-mass spectrometry; M, male(s); malocc, malocclusion; Mand, mandibular; min, minute(s); MMP, matrix metalloproteinase; mo, month(s); MS, mass spectrometry; Mx, maxillary; NS, no statistically significant difference; OIRR, orthodontically induced root resorption; OPG, osteoprotegerin; OPN, osteopontin; OTM, orthodontic tooth movement; P1NP, procollagen type 1 N-terminal propeptide; PP, dentin phosphophoryn (alternate abbreviation in the literature); pts, patients; qMS, quadrupole mass analyzer; RANKL, receptor activator of nuclear kappa B ligand; retr, retraction; RR, root resorption; SDS-PAGE, Sodium dodecyl-sulfate polyacrylamide gel electrophoresis; Sig., significant; TNF-α, tumor necrosis factor-α; TOF, time of flight; TRAP, tartrate-resistant acid phosphatase; Tx, treatment; vs, versus; wk, week(s); y, year(s).

#### Participant characteristics

The majority of studies had 20 or fewer participants. Three studies mentioned participants or teeth in two experimental and one control group with 20 patients in each group.^7^,^13^,^19^ Mah and Prasad mentioned two resorption groups; one group was examined for orthodontic resorption severity at 1–3 mm, while the second group looked at physiologic resorption of primary resorbing molars.^13^ Balducci et al. classified the two experimental groups as mild (≤2 mm) and severe resorption (>2 mm) groups,^7^ and George and Evans defined mild resorption (≤ 2 mm and severe resorption as >2 mm in their groups.^19^ A total of 9 studies examined the resorption severity grades measured in mm, or classified it as mild/moderate/severe, or as coronal/apical resorption.^5^,^7^,^9^,^12^,^13^,^19^,^20^,^24^,^29^ Resorption with respect to the duration of orthodontic treatment was considered in 7 studies.^7^,^9^,^19^,^20^,^23^,^27^,^30^

While the majority of studies had both male and female participants, two studies investigated only female participants.^9^,^15^ Most of the studies collected biomarkers for the experimental or control teeth from the maxillary central and lateral incisors. However, controls varied, depending on the study: for example, external (in different subjects),^5^,^7^,^9^,^12^,^13^,^19^,^20^,^24^,^25^,^27^,^29^,^30^ or internal (baseline values),^22^ antagonistic teeth,^15^ and contralateral teeth,^16^,^21^,^26^ while one of the studies mentioned no control.^28^ Only five studies considered physiological resorption of the primary resorbing molars.^5^,^13^,^16^,^21^,^29^

#### Study characteristics

The majority of studies were cross-sectional, although six mentioned the collection of samples at more than one observation time.^5^,^24^,^26^–^28^,^30^ There were four split-mouth design studies,^15^,^21^,^22^,^26^ two of which considered 100 g force retraction on one side of the mouth and 150 g force on the other side.^15^,^22^ The amount of resorption was judged radiographically in most studies, with intraoral periapical radiograph specified in six,^7^,^15^,^20^,^22^,^24^,^28^ panorex in three,^5^,^12^,^27^ and micro-computed tomography in only one study.^26^

#### Type of biomarkers

Dentinal proteins were examined in most of the studies, while cytokines were the focus of six studies,^9^,^16^,^17^,^19^,^26^,^29^ enzymes in two,^15^,^22^ and metabolites in one study.^27^ Of the various dentinal proteins, DSPP^2^,^25^,^29^,^30^ and DSP^5^,^7^,^16^,^23^ were studied in four studies each. However, dentinal proteins DPP,^7^ and dentin matrix protein-1 (DMP1)^13^ were studied in one study each. Pro-inflammatory cytokines, primarily interleukins (IL-1β, 2, 4, 5, 6, 7, 8, 10, 12, 13), were examined in three studies,^9^,^16^,^26^ and the interleukin-1 receptor antagonist (IL-1RA) in two studies.^16^,^29^ Receptor activator of nuclear factor kappa-B ligand (RANKL) and osteoprotegerin (OPG) were looked at in two studies^16^,^19^ and osteopontin (OPN) and tumor necrosis factor-α in one study each.^19^,^26^ Enzyme ALP was examined in two studies.^15^,^22^ Tartrate-resistant acid phosphatase (TRAP),^22^ aspartate aminotransferase (AST),^22^ and matrix metalloproteinase (MMP-8)^16^ were examined in only one study each. Cytokine profile and resorption proteins were evaluated in four studies.^12^,^20^,^21^,^28^

#### Medium and technique of biomarker evaluation

Biomarkers were evaluated in varied biofluids: the majority of samples were collected from the GCF (*n*=17 studies), saliva was used in two studies,^24^,^27^ and only Yashin et al. evaluated biofluids collected from both saliva and blood.^12^ Periopaper was used to collect GCF in 11 studies; however, other studies used micro-pipettes,^5^,^25^ filter paper,^2^,^23^ absorbent paper,^20^ and endodontic paper points.^29^ Various methods were used for evaluating biomarkers; the majority of studies used enzyme-linked immunosorbent assay (ELISA), but some studies used sodium dodecyl sulfate-polyacrylamide gel electrophoresis (SDS-PAGE),^7^,^19^,^20^ western blotting,^5^,^7^,^19^,^24^ multiplex-bead immunoassay,^26^ spectroscopy,^24^,^30^ liquid chromatography,^21^,^28^ spectrophotometry,^15^,^22^ and mass spectrometry.^21^,^28^

#### Upregulation or downregulation of biomarkers

The amount of DPP in the GCF was found to be significantly higher in resorbing primary molars (11.7±4.1 μg/mg) and orthodontically treated teeth (9.3±4.7 μg/mg) compared to the controls (5.4±4.1 μg/mg).^13^ Kereshanan et al. also showed increased DSP in the GCF of physiologically resorbing molars compared to non-resorbing teeth but no difference in coronal and apical sites of resorption.^5^ Other biomarkers such as the dentinal proteins DMP1,^7^ DPP/PP,^7^ DSP,^7^ DSPP,^29^ cytokines IL-6,^9^ RANKL,^19^ and RANKL/OPG ratio^19^ showed better GCF detection in root resorption than in controls. But IL-1RA had higher levels in the controls versus the resorption group.^29^ Studies evaluating the difference in resorption severity showed higher levels of DSP,^7^ lower levels of granulocyte-macrophage colony-stimulating factor,^26^ and higher resorption protein concentrations in severe versus mild resorption (0.89 μg/μL ±0.32 μg versus 0.77 μg/μL ±0.21 μg, respectively).^19^ Additionally, in comparison to non-resorbing teeth, mild and severe resorption showed higher RANKL/OPG ratios.^19^ Specific protein bands in saliva have also been identified in mild to moderate resorption.^20^ In physiologic root resorption of primary molars, upregulation of 37 resorption proteins was seen, as well as downregulation of 59 resorption proteins and IL-1RA levels, compared to no resorption groups in permanent molars.^16^,^21^

A few longitudinal studies evaluated the rise or fall of biomarkers in GCF with resorption at different observation times. Kereshanan et al. mentioned the rise of DSP levels in GCF after the start of fixed orthodontic treatment compared with before treatment initiation,^5^ while Thalanany et al. showed a significant increase in DSPP after two months of intrusion.^25^ Protein abundance was also evaluated in GCF by Mohd Nasri et al.^28^ In comparison, Ahuja et al. evaluated multiple markers that peaked at different observation times: IL-1β at 1 and 7 days, IL-4 at 1 and 3 days, interferon gamma (IFN-γ) at day 3, tumor necrosis factor-α at 3 hours and at 28 days, and IL-7 and granulocyte-macrophage colony-stimulating factor (GM-CSF) at 28 days.^26^

Two studies evaluated the efficacy of one method over another in the detection of biomarkers. Sha et al. found that DSPP detection by spectrophotometric ELISA (limit, 5.0 pg/mL) was less sensitive than electrochemical detection (limit, 0.5 pg/mL).^2^ Lombardo et al. showed a modified micro-bead approach to be better than standard ELISA for DSP detection in GCF.^23^

However, for salivary detection, Yashin et al. showed a significant increase in IL-7, IL-10, IL-12p70, and IFN-γ, and a significant decrease in IL-4 in moderate to severe resorption compared to controls.^12^ That same study also showed lower osteocalcin in the blood for resorption compared to no resorption.^12^ Salivary proteins have been shown to vary in young and adult root resorption groups, with an increased expression of 244 proteins in the moderate-to-severe young resorption group and only 58 proteins in the adult group compared to controls.^24^ Additionally, 187 metabolites were identified by Zhou et al. in the saliva of root resorption groups compared to their no resorption group.^27^

Cut-off values of biomarkers in orthodontic root resorption were studied by Mandour et al. at less than 432.6 pg/mL for IL-1RA and greater than 7.33 pg/mL for DSPP, with greater reliability for DSPP than IL-1RA.^29^ Additionally, Zain et al. proved that treatment duration was a contributing factor for resorption, with the absorption spectrum of DSPP rising in subjects within 3, 6, and 12 months of treatment.^30^ Studies have also evaluated changes in biomarkers in resorption associated with two different force levels (100 g and 150 g).^15^ Wahab et al. showed a statistically significant increase in TRAP levels from baseline to 3–5 weeks for 100 g force and in AST at 5 weeks for 150 g force, with the ALP group only showing a slight increase in both force levels.^22^

## DISCUSSION

**Figure 3:**
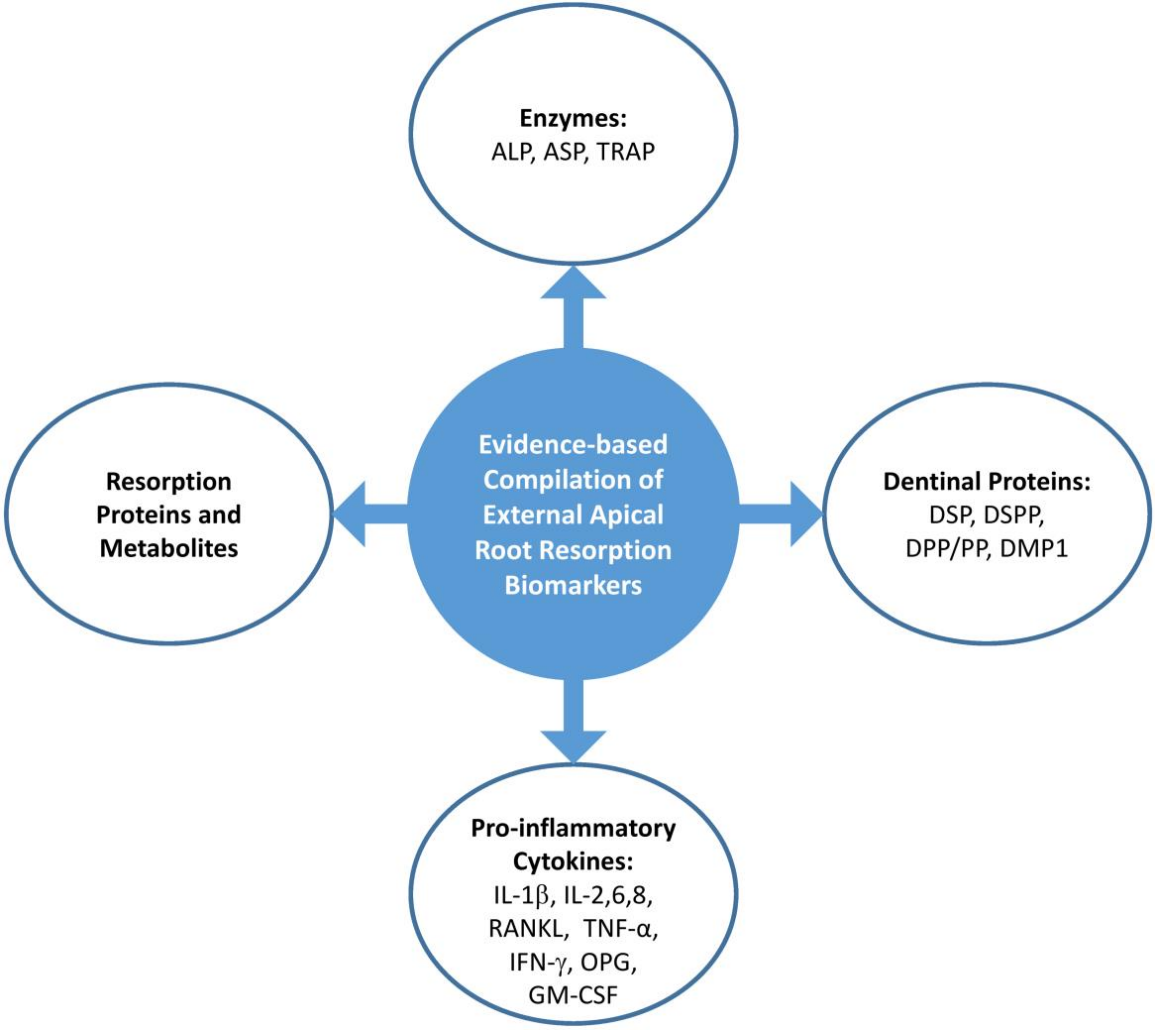
Evidence-based Compilation of Biomarkers in External Apical Root Resorption (EARR). ALP, alkaline phosphatase; AST, aspartate transaminase; DMP1, dentin matrix protein 1; DSP, dentin sialoprotein; DPP, dentin phosphophoryn; DSP, dentin sialoprotein; DSPP, dentin sialophosphoprotein; GM-CSF, granulocyte-macrophage colony-stimulating factor; IFN-γ, interferon gamma; IL, interleukin; IL-1β, interleukin-1β; OPG, osteoprotegerin; OPN, osteopontin; PP, dentin phosphophoryn (alternate abbreviation in the literature; RANKL, receptor activator of nuclear factor kappa-B ligand; TNF-α, tumor necrosis factor-α; TRAP, tartrate-resistant acid

The variation in multiple biomarkers in EARR based on the outcome measurements of severity, physiologic resorption, and orthodontic treatment versus controls, different time intervals, and methods of detection is presented in Table 3. Figure 3 presents a pictorial compilation of all biomarkers studied in this review.

**Table 3:** Evidence-based Compilation of Biomarkers in External Apical Root Resorption.

Biomarker Category	Biomarkers Studied	References to Related Studies	Specified Relative Risk Characteristics	Outcomes Related to Characteristics
Dentinal proteins	DSPP, DSP, DPP, DMP1	2, 5, 7, 13, 16, 23, 25, 29, 30	RR severity	PP and DSP in severe RR (>2 mm) greater than mild root resorption (≤2 mm)^7^ DSP in coronal RR greater than apical RR (NS)^5^
			Physiologic relative risk	DPP in primary resorbing molar greater than orthodontically treated tooth^13^ DSP levels in physiologic RR greater than non-resorbing teeth^5^
			Comparison with controls undergoing no orthodontic Tx or no relative risk	DPP in orthodontically treated teeth (1–3 mm of resorption) greater than in controls (Sig. diff)^13^ DMP1, PP, DSP in RR greater than in control groups (Sig. diff)^7^
			Time-related changes	DSP levels increased in GCF in 12 months of orthodontic treatment^5^ DSPP levels increased significantly in GCF in 2 months of intrusion compared to baseline^25^
			ELISA method for detection	DSPP detection with ELISA using spectrophotometry and electrochemistry possible but NS. Lower end of detection is 10 times greater in spectrophotometry (5 pg/mL) than in electrochemical detection (0.5 pg/mL), hence latter method more sensitive^2^ Modified micro-bead approach is more reliable than standard ELISA for DSP^23^
Cytokines and growth factors	IL (1β, 2, 4, 5, 6, 8, 10, 12, 13), TNF-α, IL-1RA, IFN-γ, OPG, OPN, RANKL, GM-CSF, salivary cytokine profile	9, 12, 16, 19, 26, 29	Severity of relative risk	RANKL/OPG ratio in severe (>2 mm) RR greater than in controls (Sig. diff)^19^ Higher GM-CSF levels in low vs high RR^26^ DSPP levels lower in controls vs orthodontic patients, and least in pediatric patients; Sig. diff between IL-1RA and DSPP, IL-1RA cut-off for OIRR (≤432.6 pg/mL) and DSPP (≥7.33 pg/mL); reliability of DSPP (100%) vs IL-1RA (80%)
			Variation with orthodontic force levels	TNF-α in GCF significantly increased in teeth receiving 225 g of controlled buccal tipping force, as early as 3 h and at 28 days when compared with contralateral control teeth^26^
			Comparison to controls with no orthodontic Tx or no relative risk	OPN (66 kDa), OPG (30 kDa) detected in RR; proteins detected in controls^19^ RANKL in root resorption greater than controls^19^ GCF IL-6 levels in female subjects with severe RR (>⅓ root) higher than without RR^9^ Moderate to severe RR shows significantly increased IL-7, IL-10, IL-12p70, and IFN-γ vs no resorption^12^ Moderate to severe RR shows significantly decreased IL-4 vs no RR^12^ In blood, RR has higher osteocalcin and P1NP vs no RR^12^
			Physiologic resorption	IL-1RA significantly downregulated in physiologic RR vs no RR^16^ IL-1RA levels greater in controls than in physiologic RR group^29^
			Early detection of biomarkers	Detection of TNF-α as early as 3 h in GCF in RR^26^
Enzymes	ALP, AST, TRAP, MMP-8	15, 16, 31	Variation with orthodontic force levels/type	ALP shows higher levels in 1 week upon application of continuous 150 g force compared to 100 g force and faster canine movement with no RR^15^ Significant increase in TRAP from baseline to 3–5 weeks in 100 g force while AST increased in 5 weeks upon application of 150 g force; 100 g force as effective as 150 g force^22^
			Early detection of biomarkers	ALP detected as early as 1 week after 150 g force application in RR patients^15^
Resorption proteins and metabolites	Protein profile in GCF and saliva	20, 21, 24, 27, 28	Comparison to controls with no orthodontic Tx or no relative risk	Higher protein bands in mild to moderate RR as compared to controls^20^ 187 salivary metabolites identified in female RR patients compared to controls^27^
			Physiologic resorption	37 RR proteins upregulated and 59 RR proteins downregulated in primary molar physiologic RR compared to teeth with no RR^21^
			Influence of age on relative risk and protein levels	In moderate-to-severe young RR group, 244 salivary proteins significantly increased and 97 decreased^24^ In moderate-to-severe adult RR group, 58 salivary proteins significantly increased and 198 significantly decreased^24^ In young mild RR group, 318 salivary proteins significantly increased and 78 decreased^24^ In adult mild RR group, 102 salivary proteins increased, and 153 significantly decreased^24^
			Potential biomarker candidates	Fetuin-A and p21-ARC^24^
			Early detection of biomarkers	Early increase of 16 proteins in GCF in mild RR patients after 1 month of orthodontic force application^28^

ALP, alkaline phosphatase; AST, aspartate aminotransferase; DMP1, dentin matrix protein 1; DPP, dentin phosphophoryn; DSP, dentin sialoprotein; DSPP, dentin sialophosphoprotein; ELISA, enzyme-linked immunosorbent assay; GCF, gingival crevicular fluid; GM-CSF, granulocyte-macrophage colony-stimulating factor; h, hours; IFN-γ, interferon gamma; IL, interleukin; IL-1RA, interleukin-1 receptor antagonist; MMP-8, matrix metalloproteinase-8; NS, not significant; OIRR, orthodontically induced root resorption; OPG, osteoprotegerin; OPN, osteopontin; P1NP, procollagen type I N-terminal propeptide; p21-ARC, cyclin-dependent kinase inhibitor p21; PP, dentin phosphophoryn (alternate abbreviation in the literature); RANKL, receptor activator of nuclear kappa B ligand; RR, root resorption; Sig. diff, significant difference; Tx, treatment; TNF-α, tumor necrosis factor-α; TRAP, tartrate-resistant acid phosphatase.

Wide heterogeneity was noticed in the reviewed studies with regard to tooth selection for resorption, study settings, biomarker selection, collection, and evaluation. However, the majority of studies took measures to alleviate confounding bias in terms of inflammation caused by coexistent periodontal or gingival inflammation. Several studies ensured good oral hygiene and gingival and periodontal condition by measuring probing depth, bleeding on probing, and the gingival index, since inflammation may alter the biomarker levels in biofluids.^2^,^5^,^7^,^9^,^12^,^13^,^15^,^16^,^20^,^21^,^23^,^24^,^26^–^28^,^30^,^31^ Furthermore, to rule out confounding variables for biomarker levels, many of the studies excluded patients with smoking, pregnancy, previous orthodontic treatment or systemic illness, and craniofacial disorders. A few studies also mentioned discouraging the use of antibiotics and anti-inflammatories^7^,^9^,^13^,^15^,^20^,^22^,^23^,^27^,^28^ or mouthwashes like chlorhexidine,^22^ but this was not a standard practice across all the studies.

Various biomarkers in the GCF were identified by this review, including dentinal proteins (DPP, DSP), cytokines (IL-6, OPG, OPN), RANKL, and enzymes (ALP, AST). A few of these were identified in the 2019 systematic review by Tarallo et al.,^17^ who evaluated EARR biomarkers in GCF from seven studies after quality assessment. However, this scoping review identified additional biomarkers, including DSPP, DPP, DMP1, cytokines, and their receptor antagonists (IL-1β, 2, 4, 5, 6, 7, 8, 10, 12, 13, TNF-α, OPG, OPN, RANKL, and IL-1RA), along with resorption proteins in both the GCF and saliva. A recent review by Mona et al. evaluated protein–protein interactions of EARR biomarkers in variable study designs of human and animal studies, including case-control studies, reviews, and physiologic resorption.^32^ However, it has limited applicability in studying resorption in clinical orthodontic practice, unlike this scoping review. In light of these data, future research should include a bioinformatics analysis for the biomarkers identified by this scoping review, to ascertain the protein interactions responsible for clinical resorption overlapping other periodontal and pathological problems.

The majority of studies in the current review identified dentin-specific proteins in EARR, especially DSPP, DSP, DPP/PP, and DMP1. Of these, the DSP and DPP proteins are the most abundant non-collagenous proteolytic cleavage products of DSPP found in dentin (5%–8% and 50%, respectively). This review also identified DSP as a potent resorption marker,^5^,^7^,^16^,^23^ both in orthodontic and physiologic resorption.^2^,^5^ It is more dentin-specific than DPP and is found in odontoblasts and the extracellular matrix of pre-dentin, dentin, and dental pulp, but is not prevalent in bone, cartilage, ameloblasts, or other oral tissue components.^5^ However, the presence of DSP and DPP in control subjects with no resorption^5^,^13^ also indicates the release of dentinal matrix proteins in the GCF from pulpal cells during root mineralization in young permanent teeth with patent apices. These dentinal matrix proteins may not be exclusively present in dentin, since both are products of a larger precursor protein, DSPP, which is also present in osteoblast cells.^5^ Osteopontin is another glycosylated protein of the dentin matrix and bone, produced by odontoblasts along with other bone precursors such as cementum and macrophages. The current review shows the presence of degraded fragments (54 kDa and 66 kDa) of OPN in the GCF of mild and severe resorption.^19^ This occurs as a result of the enzymatic activity of cysteine proteases, causing degradation of bone and the dentin extracellular matrix, which is also seen in periodontal disease.^33^ In addition, this review found that different cytokines, including pro-resorptive IL-6, show higher GCF levels in severe compared to no resorption,^9^ which is supported by rat studies showing an association of IL-6 with induction and further progress of mechanically induced root resorption.^34^ Furthermore, IL-6 has an established role in osteoclastogenesis and bone remodeling associated with orthodontic force application by inducing RANKL and osteoclasts formation.^35^ Additionally, osteoclastogenesis is governed by the RANKL/OPG ratio,^10^ as seen in the current review, where this ratio was significantly higher in severe resorption than in controls.^19^ Other clastogenic mediators (TNF-α and IL-7) also augment resorption in GCF,^26^ with previous literature supporting their role in bone resorption in orthodontic tooth movement.^10^

The orthodontic force levels, 150 g force versus 100 g force, seem to have no effect on tooth resorption. Nevertheless, 150 g force application causes a significant increase in ALP on the mesial side within one week compared to 100 g force.^15^ Alkaline phosphatase (ALP) is known to support osteoblastic activity.^11^ A similar rise in ALP was seen in previous studies at 1 to 3 weeks^36^ and at 2 weeks after orthodontic force application.^37^ The TRAP and AST enzymes also vary with the level of force. The TRAP levels showed a significant rise from baseline with 100 g force but not with 150 g force.^22^ The AST on the other hand showed a significant rise with 150 g force within 5 weeks, but not with 100 g force.^22^ Previous literature also supports a rise in TRAP proportionally with the orthodontic force magnitude,^31^ and higher AST levels at compression versus tension sites, thus favoring the resorptive activity.^38^

This review found that salivary metabolome was associated with specific clusters of metabolites in EARR using partial least squares discriminant analysis, which may be further explored for diagnosis of resorption.^27^ These clusters include purine and arachidonic acid metabolites, known for chemotaxis of inflammatory cells as well as periodontal damage propagation/resorption.^39^ This further produces reactive oxygen species causing a shortage of local oxygen concentrations, and triggering the RANKL pathway.^39^ Thus, these metabolites may indicate resorption as well as periodontal damage, further confirming the need to ascertain periodontal health when performing such biomarker studies or examining the reciprocal effect of periodontal inflammation on these biomarkers and on resorption.

Best practices for biomarkers isolation and detection have also been highlighted by this review. While several of the reviewed studies primarily mentioned conventional ELISA, two comparative studies established the increased sensitivity of electrochemical over spectrophotometric ELISA,^2^ as well as micro-beads over conventional ELISA.^23^ These conventional and microbead assays offer several advantages: they are sensitive, non-invasive, include no radiation exposure, provide stage-wise monitoring and at-risk assessment, and can be used to diagnose and predict the clinical course of therapy.^13^ This review also found a newer non-invasive approach for non-targeted metabolomics using high-resolution nuclear magnetic resonance spectroscopy.^27^ This method can identify newer mediators or varied human disease pathways in the EARR domain, offering significant benefits by providing multi-component information simultaneously.

Hence, the current review answers our primary research question by examining the variation in levels of all biomarkers in EARR which can be isolated in the oral fluids. The resorption markers have been studied in orthodontic treatment as well as in comparison with physiologic resorption. In addition, this review also highlights the best methods for biomarker isolation. It also mentions the study design drawbacks for consideration in future evaluations and proposes further bioinformatic analysis of identified cellular markers.

## LIMITATIONS

Although the reviewed studies met all inclusion and exclusion criteria, there was an extensive heterogeneity of biomarkers, including a wide range of cytokines, dentinal proteins, receptors, and colony-stimulating factors, as well as resorptive proteins and metabolites. The study designs were also varied, mostly cross-sectional using single observation samples, although a few studies evaluated resorption longitudinally with variation in mediator levels at different time points. None of the reviewed studies performed randomization to examine the effects of variable orthodontic forces or treatments on resorption. The sample size was generally small and unequal between the experimental and control groups in the majority of studies. Other confounders were unequal male-to-female ratio, no standardization of study prerequisites related to inflammatory conditions or history of smoking, and antibiotics or anti-inflammatories, all of which may have a bearing on biomarker levels.

## CONCLUSIONS

The conclusions of this scoping review may be summarized as follows:

- Several biological markers have been identified in external apical root resorption in various oral body fluids (GCF, saliva, and blood). These include dentinal proteins, cytokines, enzymes, and protein metabolites.
- Dentinal proteins (DSP,^7^ DMP1,^7^ DPP/PP,^7^,^13^ and DSPP^29^,^30^) and cytokines (IL-6,^9^ IL-1β,^26^ IL-4,^26^ TNF-α,^26^ IFN-γ,^26^ RANKL,^19^ and RANKL/OPG ratio^19^) show significant increase, and granulocyte-macrophage colony-stimulating factor^26^ levels decrease in resorption compared to no resorption. The opposite is true for IL-1RA which is higher in controls.^16^,^29^
- Physiologically resorbing teeth show higher DSP,^5^ DPP/PP,^13^ and DSPP^29^ and lower IL-1RA levels^16^ when compared with non-resorbing permanent teeth.
- Higher severity of resorption showed increased DSP,^7^ DPP,^7^ and RANKL/OPG ratio^19^ and higher resorption protein concentration^19^ compared to mild resorption, although the evidence is scanty.
- Salivary biomarkers show significant increase in IL-7,^12^ IL-10,^12^ IL-12p70,^12^ IFN-γ,^12^ resorption proteins,^24^ and metabolites^27^ and significant decrease in IL-4^12^ in resorption.
- Cut-off values of biomarkers for root resorption were mentioned with IL-1RA (<432.6 pg/mL) and DSPP (>7.33 pg/mL), but this evidence requires further validation.^29^
- Detection of DSPP by electrochemical ELISA (limit, 0.5 pg/mL) is more sensitive than spectrophotometric ELISA (limit, 5.0 pg/mL).^2^ Furthermore, DSP detection in the GCF by modified micro-bead approach proved better than standard ELISA.^23^

Several points for further investigation are suggested based on the findings of the current review:

- Next steps include identifying the most sensitive and specific biomarkers (dentinal proteins/inflammatory cytokines/metabolites) in the GCF or saliva for early-stage EARR detection, and evaluating them repeatedly during the progress of treatment. A biosensor point-of-care screening device based on the most potent biomarker to detect root resorption is also suggested.^40^
- Cut-off levels for biomarkers need to be established, and a non-invasive clinical test developed for early diagnosis of iatrogenic resorption.
- Study designs should be standardized to generate unbiased high-quality evidence.
- Bioinformatic analysis is needed to identify the protein interactions, which may also overlap with other oral inflammatory conditions including external cervical resorption in chronic periodontitis.^41^

## Abbreviations

ALP: alkaline phosphatase
AST: aspartate aminotransferase
DMP1: dentin matrix protein 1
DPP: dentin phosphophoryn
DSP: dentin sialoprotein
DSPP: dentin sialophosphoprotein
EARR: external apical root resorption
ELISA: enzyme-linked immunosorbent assay
GCF: gingival crevicular fluid
GM-CSF: granulocyte-macrophage colony-stimulating factor
IFN-γ: interferon gamma
IL: interleukin(s)
IL-1RA: interleukin-1 receptor antagonist
MMPs: matrix metalloproteinases
OPG: osteoprotegerin
OPN: osteopontin
PP: phosphophoryn (alternate abbreviation in the literature)
PRISMA: Preferred Reporting Items for Systematic Reviews and Meta-Analysis
PRISMA-ScR: PRISMA extension for Scoping Reviews
RANKL: receptor activator of nuclear factor kappa-B ligand
RR: root resorption
TRAP: tartrate-resistant acid phosphatase.
